# Consumption of Yogurt and the Incident Risk of Cardiovascular Disease: A Meta-Analysis of Nine Cohort Studies

**DOI:** 10.3390/nu9030315

**Published:** 2017-03-22

**Authors:** Lei Wu, Dali Sun

**Affiliations:** 1 Department of Epidemiology, Institute of Geriatrics, Chinese People’s Liberation Army General Hospital, Beijing 100853, China; 2 Department of Nanomedicine, Houston Methodist Research Institute, Houston, TX 77030, USA; samio4762@gmail.com

**Keywords:** yogurt intake, stroke, coronary heart disease, cardiovascular disease, meta-analysis

## Abstract

Previous systematic reviews and meta-analyses have evaluated the association of dairy consumption and the risk of cardiovascular disease (CVD). However, the findings were inconsistent. No quantitative analysis has specifically assessed the effect of yogurt intake on the incident risk of CVD. We searched the PubMed and the Embase databases from inception to 10 January 2017. A generic inverse-variance method was used to pool the fully-adjusted relative risks (RRs) and the corresponding 95% confidence intervals (CIs) with a random-effects model. A generalized least squares trend estimation model was used to calculate the specific slopes in the dose-response analysis. The present systematic review and meta-analysis identified nine prospective cohort articles involving a total of 291,236 participants. Compared with the lowest category, highest category of yogurt consumption was not significantly related with the incident risk of CVD, and the RR (95% CI) was 1.01 (0.95, 1.08) with an evidence of significant heterogeneity (*I*^2^ = 52%). However, intake of ≥200 g/day yogurt was significantly associated with a lower risk of CVD in the subgroup analysis. There was a trend that a higher level of yogurt consumption was associated with a lower incident risk of CVD in the dose-response analysis. A daily dose of ≥200 g yogurt intake might be associated with a lower incident risk of CVD. Further cohort studies and randomized controlled trials are still demanded to establish and confirm the observed association in populations with different characteristics.

## 1. Introduction

Cardiovascular disease (CVD) is still an important public health problem around the world [1]. The prevalence of coronary heart disease (CHD) and stroke has progressively increased during the past decades [1,2,3]. Given the very large social and economic burden of the treatment of CVD [4], identifying modifiable factors is imperative and feasible for preventing the progress of CVD.

Several dietary patterns and individual foods have been demonstrated to exert preventive effects on CVD risk [5,6,7]. In particular, the benefits of yogurt intake have recently drawn a lot of attention [8,9]. Yogurt is defined as the product of fermentation of the *Lactobacillus delbrueckii* subspecies *bulgaricus* and *Streptococcus thermophilus* [10]. Being an important component of the human diet for several millennia [10], it will be a major public health implication if yogurt consumption is demonstrated to have a protective role in delaying the development of CVD.

Previous systematic reviews and meta-analyses have evaluated the association of dairy consumption and the risk of CVD [11,12,13,14,15]. However, the conclusions were inconsistent. In the subgroup analysis of yogurt intake, no association was established between yogurt intake and CVD or stroke in three previous meta-analyses [11,12,13]. Soedamah-Muthu et al. did not separately estimate the effect of yogurt intake apart from other dairy products [14]. Hu et al. observed a protective role of yogurt intake on stroke, but only three studies were included in the pooled analysis [15].

To the best of our knowledge, no quantitative analysis has specifically assessed the effect of yogurt intake on the risk of incident CVD. Moreover, whether different amounts of yogurt consumption present different impacts on CVD risk is still uncertain. Therefore, we performed a systematic review and meta-analysis to pool the evidence from prospective cohort studies on the relationship of yogurt intake and the incident risk of CVD. Furthermore, we attempted to evaluate the potential dose-response pattern of the association.

## 2. Materials and Methods

### 2.1. Literature Search

The present systematic review and meta-analysis was carried out according to a standard process [16,17]. We searched the PubMed and the Embase databases from inception to 10 January 2017 for records relevant to yogurt consumption and risk of incident CVD. Our search included terms as “fermented dairy”, “yogurt”, “yoghurt”, “sour milk”, “fermented milk”, “cultured milk”, “probiotic”, etc. Language restriction was not set. A detailed search strategy is presented in Supplementary Table S1. The references of the relevant reviews and original articles were manually searched to find out more potential eligible studies. When multiple published articles were found from an identical study, the one with the longest follow-up duration was included in the present analysis.

### 2.2. Selection Criteria and Data Extraction

The authors independently conducted the initial screening process. After removing the duplicate records, we identified the title and the abstract of each eligible article. Unrelated articles were excluded, and articles of interest were included as further evaluation. Any disagreements were resolved by discussion between the two authors.

Inclusive criteria: (1) studies reporting the relationship of yogurt intake and the incident risk of CVD (CHD or stroke) by using adjusted relative risks (RRs), hazard ratios (HRs), or odds ratios (ORs) and their corresponding 95% confidence intervals (CIs); (2) studies in which exposures were the fermented milk, but yogurt was the largest contribution to the total fermented milk; and (3) the study design was based on prospective cohort. Exclusive criteria: (1) the data reported individual components of yogurt, such as protein or probiotics; (2) studies that only reported results for total dairy/milk products or combined non-fermented and fermented milk; (3) the studies in which the participants are aged <18 years; and (4) the studies in which the participants are pregnant or lactating females.

Data extraction was independently implemented by two authors. Data were extracted from each eligible article including the first author, the published year, the study location, the number of cases and total participants, baseline age and gender of participants, the method of exposure and the outcome measurements, the type of exposure and outcome, duration of follow-up, adjusted variables, and the largest number of adjusted ORs, RRs, or HRs with their corresponding 95% CIs of incident CVD for all categories of yogurt consumption.

### 2.3. Quality Assessment

The Newcastle-Ottawa quality scale (NOS) [18] was used to estimate the quality assessment of all eligible articles. Higher points indicated higher study quality, and the scale ranged between 0 and 9 points. Three domains were assessed: (1) the basis of the cohort selection (0–4 points); (2) the comparability of the cohort design and analysis (0–2 points); (3) and the adequacy of measurements including exposure and outcome variables (0–3 points).

### 2.4. Statistical Analysis

Statistical analyses were conducted using two sorts of software: Stata (12.0, StataCorp LP, College Station, TX, USA) and Review Manager (5.2, The Nordic Cochrane Centre, Copenhagen, Denmark). We applied RRs to measure the effect size for articles using the incident cases of CVD as an outcome. An approach of generic inverse-variance was used to pool the outcome data for the yogurt intake of highest vs. lowest category with a random-effects model. The *p*-values less than 0.05 were regarded as statistical significant. Heterogeneity across studies was examined by the *I*^2^ statistic which, when greater than 50%, indicated significant results [19]. Additionally, we conducted a stratified analysis based on pre-specified characteristics including the type of CVD (CHD or stroke), study location (North America or Europe), age (<40 or ≥40 years), gender (male, female or both sexes), the exposure type (yogurt or combined with other dairy products), and the exposure dose (<200 or ≥200 g/day). Furthermore, meta-regression analysis was used to identify the potential difference of the two groups, *p*-values of less than 0.1 were judged as significant. To evaluate the effect of an individual article on the overall pooled results, a sensitivity analysis was conducted by omitting each article from the overall analysis in every turn. The publication bias was examined through the tests of Begg’s and Egger’s [20,21].

Generalized least squares trend (GLST) estimation model was used to compute the specific slopes in the dose-response analysis [22,23]. For categories (at least three) of yogurt consumption that were open (e.g., 30–69 g/day), we assigned the median value as the homologous category of yogurt intake. If the maximum dose was unlimitedly fixed (e.g., >200 g/day), we assumed that the mean was 25% larger than the lower level of the specific category [24]. When the number of cases for each category was not available, the RRs were acquired with a general estimate [25]. When studies reported yogurt intake in serving/day, we converted the intake to g/day using a standard unit of 244 g [26]. The results of the dose-response analysis were shown for each gram increased in daily yogurt intake. A restricted cubic spline model (four-knot) was applied for the assessment of non-linearity hypothesis in the association between yogurt intake and the incident risk of CVD. 

## 3. Results

### 3.1. Article Identification and Selection

**Figure 1:**
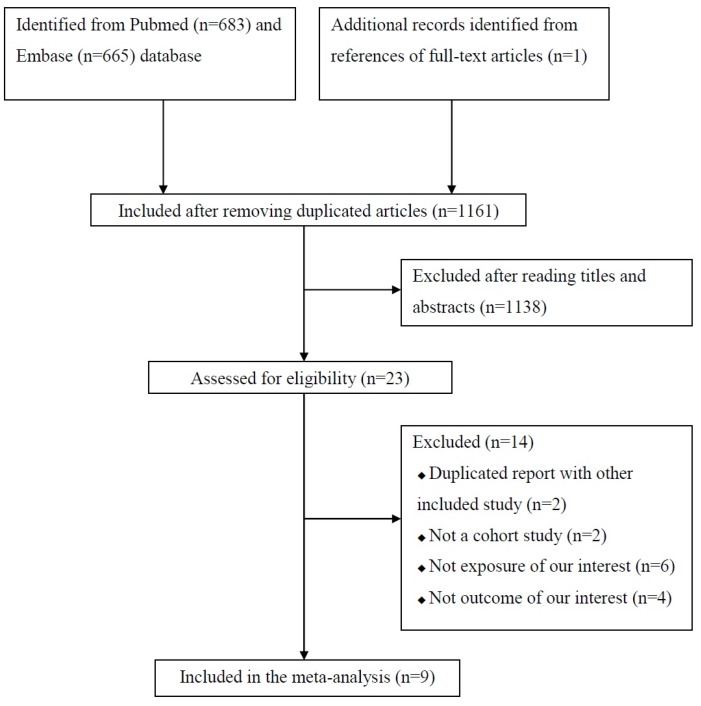
Flow diagram of articles included in the present study.

Figure 1 presents a flow diagram of articles included in the present study. In the initial search process, 1348 studies were identified from the Pubmed and the Embase databases. After removing the duplicated articles, 1161 studies were included for further assessment. A total of 1138 studies were excluded after reading the titles and the abstracts. The remaining 23 studies were evaluated to assess for eligibility after reading the full-text. Finally, nine cohort studies were eligible for inclusion in our meta-analysis [27,28,29,30,31,32,33,34,35]. One of the nine articles was identified from references of a full-text article [29].

### 3.2. Study Characteristics

Characteristics of each included article are shown in Table 1. Publication years were ranged between 1999 and 2015. Two articles were conducted in North America [27,29] and the remaining seven articles were performed in Europe [28,30,31,32,33,34,35]. Follow-up durations ranged between 10.2 [31] and 17.3 years [33]. Six articles included both men and women [27,28,31,33,34,35], one article included only men [30], and two articles included only women [29,32]. The baseline age of the participants ranged from ≥21 [28] to ≥55 years [33]. The number of study participants ranged from 1759 [27] to 85,764 [29] for a total number of 291,236. Yogurt intake was assessed by a food-frequency questionnaire (FFQ), and the incidences of CVD were ascertained from medical records or registries in all included articles.

### 3.3. Quality Assessment

All studies received a quality score of 8–9 stars (Supplementary Table S2). All articles measured yogurt intake by FFQ. The diagnoses of CVD were ascertained from medical records or registries in all included articles. The follow-up duration of all articles was greater than 10 years. One article did not exclude participants with a history of CHD events [27], and one study only included the elderly male smokers (at a higher risk of CVD) [30]. One article only adjusted for age and smoking status in the statistical model [29].

### 3.4. Yogurt Consumption and the Occurrence of CVD

**Figure 2:**
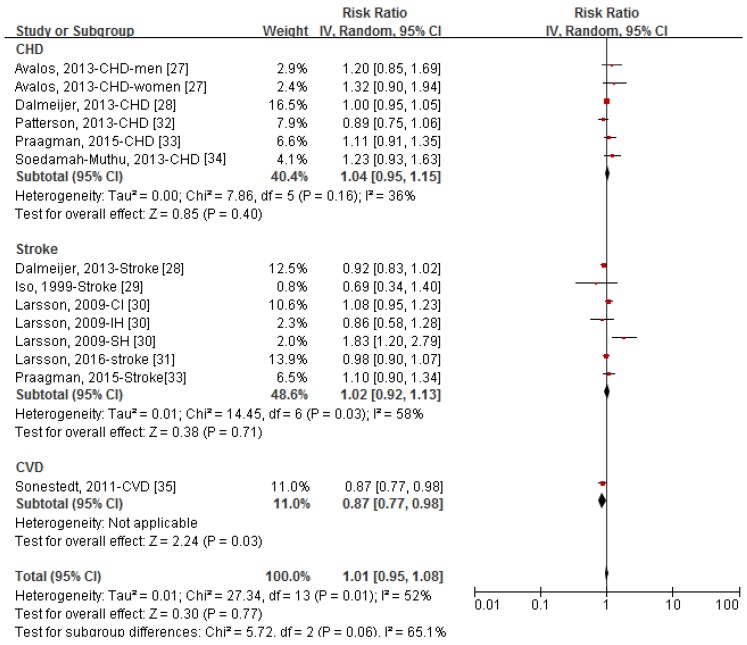
Forest plot of relative risks (RRs) and 95% confidence intervals (CIs) for the association between category of yogurt intake (highest vs. lowest) and the incident risk of coronary heart disease (CHD), stroke, and cardiovascular disease (CVD). CI, cerebral infarction; IH, intra-cerebral hemorrhage; SH, subarachnoid hemorrhage.

Figure 2 shows the forest plot of RRs (95% CIs) for the relationship of yogurt consumption (highest vs. lowest dose) and the occurrence of CVD by type of outcome. Yogurt consumption was not significantly associated with the developing of CVD in the pooled analysis of 14 comparatives, and the RR (95% CI) was 1.01 (0.95, 1.08) with an evidence of significant heterogeneity (*I*^2^ = 52%). In the stratified analysis by type of outcome, the pooled RRs (95% CIs) of yogurt consumption were 1.04 (0.95, 1.15) for CHD, 1.02 (0.92, 1.13) for stroke, and 0.87 (0.77, 0.98) for the incident CVD events. Sensitivity analysis showed that further exclusion of any individual comparative did not significantly alter the pooled RR, and the RRs (95% CIs) ranged between 0.99 (0.94, 1.07) and 1.03 (0.96, 1.09). Exclusion the study by Larsson et al. [30] reduced the heterogeneity to 40%. No publication bias was observed among 14 comparatives (Supplementary Figure S1, Egger’s test: *p*-value = 0.228, Begg’s test: *p*-value = 0.254).

### 3.5. Subgroup Meta-Analysis

As shown in Table 2, analyses by study location, age, gender, and the type of exposure did not significantly affect the associations between yogurt intake and the incident risk of CVD (*p*-value for difference >0.1 for each group). Stratified analysis by yogurt dose of the highest category (<200 or ≥200 g/day) significantly affected the association (*p*-value for difference = 0.09), and ≥200 g/day yogurt intake was significantly associated with lower risk of CVD compared with the reference category, and the RR (95% CI) was 0.92 (0.85, 1.00).

### 3.6. Dose-Response Analysis

**Figure 3:**
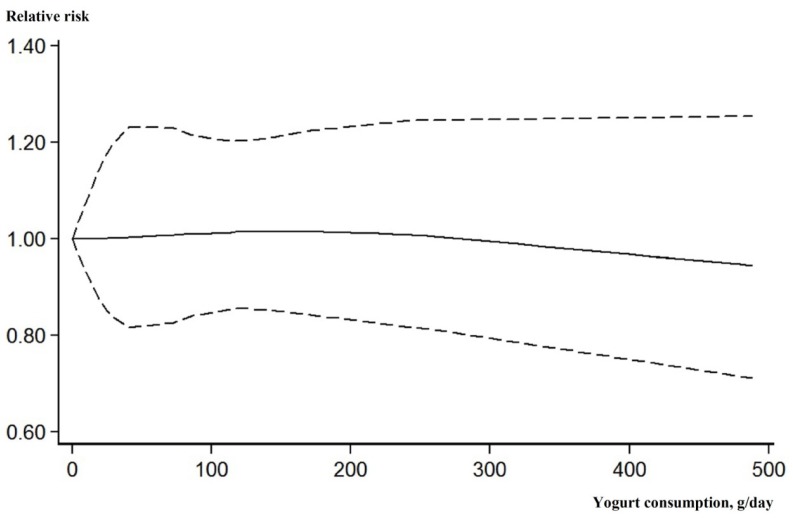
Dose-response association between yogurt consumption (g/day) and the incident risk of cardiovascular disease. Solid line, best-fitting restricted cubic spine; dotted line, 95% confidence interval.

After excluding three articles [27,28,29] that reported fewer than three categories of yogurt consumption, the remaining six studies were included in the dose-response analysis. Although the association was not significant, there was a trend that higher level of yogurt consumption was associated with a lower risk of incident CVD events (Figure 3).

## 4. Discussion

The present systematic review and meta-analysis identified nine cohort articles involving a total of 291,236 participants. Compared with the lowest category, the highest category of yogurt consumption was not significantly related with the incident risk of CVD; however, intake of ≥200 g/day yogurt was significantly associated with a lower risk of CVD in the subgroup analysis. There was a trend that a higher level of yogurt consumption was associated with a lower incident risk of CVD in the dose-response analysis.

An increasing number of epidemiological studies have supported the beneficial effects of yogurt consumption on lowering blood pressure, total cholesterol concentrations, total cholesterol, and plasma glucose [9,36,37]. Although the findings remain controversial, several epidemiological and clinical studies have suggested the potential role of yogurt intake in weight management [38,39]. Obesity, hyperlipoidemia, and high blood pressure are well-known risk factors of CVD and, thus, the above studies indirectly support the beneficial role of yogurt consumption on CVD risk. However, in agreement with several previous meta-analyses [11,12,13], we did not observe a significant association between yogurt intake and CVD. We have obtained a significantly reverse association when only including the highest yogurt consumption of ≥200 g/day, indicating that lower consumption of yogurt (<200 g/day) may represent a missed opportunity to contribute to a lower risk of CVD. Yogurt provides a good source of active components, such as calcium, vitamin D, sphingolipids, and probiotics [40]. Among these, probiotic micro-organisms have an effect on weight reduction, which may translate to a reduced risk of CVD through supporting a healthy gut microbiota composition [41]. In addition, some studies revealed that yogurt might interfere with cholesterol synthesis. However, whether the effects on lipids could be translated to a decreasing risk of CVD is warranted to confirm in the future [6].

Compared with previously published meta-analyses relevant to this topic [11,12,13,14,15], this is the first systematic review and meta-analysis to specifically evaluate the association between yogurt intake and CVD risk. All included studies were of high-quality, and the follow-up durations were long enough for outcomes to occur. Furthermore, the larger sample size (12,262 cases among 291,236 participants) enabled us to perform stratified and dose-response analyses to explore the potential association. Finally, unlike the study by Qin [11] and de Goede et al. [13], only CVD incidence, but not mortality case, was accepted as an outcome.

Limitations of our meta-analysis should be mentioned. First, considerable heterogeneity has been observed across studies. This is not surprising given the diversity in study characteristics of participants, various doses of yogurt consumption, different type of outcome, and adjusted confounders. Further sensitivity analysis revealed that the study by Larsson et al. [30] might be the source of heterogeneity. Different from other studies, participants with a higher risk of stroke were included, and three types of stroke were separately reported in the work by Larsson et al. [30]. Second, detailed data relevant to the patterns of yogurt, such as species of the lactic acid bacteria (*Lactobacillus sp., Enterococcus sp.,* or *Streptococcus sp*.), consuming time and fat-containing (skim fat, whole-fat, or low-fat yogurt) were not illustrated in all included studies. Some RCTs have indicated that intake of low-fat and whole-fat dairy products may cause different effects on blood pressure, weight and depressive symptoms [42,43]. Therefore, further studies are needed to resolve this issue. Third, only baseline dietary habits were collected and analyzed in all included studies. Participants may have changed their lifestyle and dietary patterns during the long follow-up period. Fourth, although most studies adjusted for nearly all the important covariates, other potential unmeasured confounders may have influence our findings. Fifth, all studies were conducted in Western developed countries, limiting the generalizability of the results to a broader demographic. Considering that the consumption and making methods of yogurt vary greatly from country to country [10], region-difference should be considered. Finally, our findings are based on observational studies and, thus, causal association cannot be established.

## 5. Conclusions

In conclusion, the present meta-analysis based on nine independent cohort studies provides a non-significant association between yogurt intake and CVD risk. Daily dose of ≥200 g yogurt intake might be associated with a lower risk of CVD. Further cohort studies and randomized controlled trials are still demanded to establish and confirm the observed association in populations with different characteristics.

## Figures and Tables

**Table 1:** Characteristics of the included articles.

First Author, Published Year	Study Location	Follow-Up (Years)	Male (%)	Baseline Age (Years) (Minimum-)	Participants, No.	Exposure		Outcome			Adjustment *
						Type	Category	Method of Ascertainment	Type	Case, No.	
Avalos, 2013 [27]	US	16.3	42.7	49-	1759	Yogurt	Never/rarely, sometimes/often	Medical records	CHD	454	1–6
Dalmeijer, 2013 [28]	Netherland	13.1	25.5	21-	33,625	Buttermilk, yogurt, cheese	Per SD of the mean g/day	Registries	CHD, Total stroke	1648, 531	1, 2, 7–18
Iso, 1999 [29]	US	13.6	0.0	34-	85,764	Yogurt	≥5 times/week, never	Registries and medical records	Ischemic stroke	347	1, 10
Larsson, 2009 [30]	Finland	13.6	100	50-	26,556	Yogurt	Quintile	Registries	Cerebral Infarction, Intracerebral Hemorrhage, Subarachnoid Hemorrhage	1950, 277, 114	1, 2, 8–11, 13–17, 19–29
Larsson, 2016 [31]	Sweden	10.2	53.8	45-	74,961	Yogurt, sour milk	Quintile	Registries	Total stroke	4089	1, 2, 7–11, 13–17, 22, 24, 30–32
Patterson, 2013 [32]	Sweden	11.6	0.0	48-	33,636	Yogurt	Quintile	Registries	Myocardial infarction	1392	8–11, 14, 15, 21, 24, 28, 30–34
Praagman, 2015 [33]	Dutch	17.3	37.9	55-	4235	Yogurt	<50, 50–100, >100 g/day	Registries	CHD, Total stroke	564, 567	1, 2, 7, 8, 10, 11, 13–18, 24, 35
Soedamah-Muthu, 2013 [34]	UK	10.8	72.0	35-	4255	Yogurt	Tertiles	Registries	CHD	323	1, 2, 8–11, 13–17, 24, 32, 35
Sonestedt, 2011 [35]	Sweden	12.0	38.1	44-	26,445	Fermented milk	Quartile	Registries	CVD	2520	1, 2, 7–11, 13–16, 24, 28, 36–38

* 1 = age, 2 = body mass index (BMI), 3 = diabetes, 4 = hypertension, 5 = LDL-cholesterol, 6 = estrogen use, 7 = gender, 8 = total energy intake, 9 = physical activity, 10 = smoking, 11 = education, 12 = ethanol intake, 13 = coffee intake, 14 = fruit intake, 15 = vegetables intake, 16 = fish intake, 17 = meat intake, 18 = bread intake, 19 = supplementation group, 20 = cholesterol, 21 = serum HDL cholesterol, 22 = diabetes, 23 = heart disease, 24 = alcohol intake, 25 = sugar intake, 26 = poultry intake, 27 = potatoes intake, 28 = whole grains intake, 29 = refined grains intake, 30 = aspirin use, 31 = hypertension, 32 = family history of myocardial infarction, 33 = waist-to-hip ratio, 34 = hormone therapy usage, 35 = tea intake, 36 = ethnicity, 37 = employment grade, 38 = season method. CHD, coronary heart disease; CVD, cardiovascular disease; SD, standard deviation.

**Table 2:** Stratified analysis of the association between yogurt consumption and the incident risk of cardiovascular disease.

	Comparisons, No.	Relative Risk (95% Confidence Interval)
Total	14	1.01 (0.95, 1.08)
Study location		
North America	3	1.15 (0.87, 1.52)
Europe	11	1.00 (0.94, 1.07)
*p*-value for difference		0.36
Age		
<40 years	4	0.98 (0.90, 1.08)
≥40 years	10	1.04 (0.94, 1.14)
*p*-value for difference		0.66
Gender		
Male	4	1.17 (0.92, 1.48)
Female	3	0.97 (0.72, 1.32)
Both sexes	7	0.98 (0.93. 1.05)
*p*-value for difference		0.26
Exposure type		
Yogurt	11	1.06 (0.96, 1.18)
Yogurt combined with other dairy products	3	0.96 (0.89, 1.04)
*p*-value for difference		0.25
Dose of the highest category		
<200 g/day	11	1.06 (0.98, 1.15)
≥200 g/day	3	0.92 (0.85, 1.00)
*p*-value for difference		0.09

## Supplementary Materials

The following are available online at http://www.mdpi.com/2072-6643/9/3/315/s1, Figure S1: Funnel plot of the 14 comparatives, Table S1. Search strategy, Table S2: Study quality of each included article (maximum: 9 stars).
