# Supplementary material for: Consumption of Yogurt and the Incident Risk of Cardiovascular Disease: A Meta-Analysis of Nine Cohort Studies
Source: Nutrients. 2017 Mar 22;9(3):315. doi: 10.3390/nu9030315 (PMC5372978; doi:10.3390/nu9030315)
Supplement: Supplementary file 1 [file nutrients-09-00315-s001.docx]

| **Table S1.** Search strategy. |
| --- |
| Source: PubMed and Embase (Searched on: January 10th, 2017) |
| #1 fermented dairy  #2 yogurt  #3 yoghurt  #4 sour milk  #5 fermented milk  #6 cultured milk  #7 probiotic  #8 acute coronary syndromes  #9 myocardial infarction  #10 coronary artery disease  #11 coronary heart disease  #12 coronary disease  #13 ischemic heart disease  #14 cardiovascular disease  #15 stroke  #16 angina  #17 heart attack  #18 cerebrovascular*  #19 #1 OR #2 OR #3 OR #4 OR #5 OR #6 OR #7  #20 #8 OR #9 OR #10 OR #11 OR #12 OR #13 OR #14 OR #15 OR #16 OR #17 OR #18  #21 #19 AND #20 |

| **Table S2****.** Study quality of each included article (maximum: 9 stars) | | | | |
| --- | --- | --- | --- | --- |
| First author, published year | Selection | Comparability | Outcome | Total |
| Avalos, 2012 | *** | ** | *** | ******** |
| Dalmeijer, 2013 | **** | ** | *** | ********* |
| Iso, 1999 | **** | * | *** | ******** |
| Larsson, 2009 | *** | ** | *** | ******** |
| Larsson, 2012 | **** | ** | *** | ********* |
| Patterson, 2012 | **** | ** | *** | ********* |
| Praagman, 2015 | **** | ** | *** | ********* |
| Soedamah-Muthu, 2013 | **** | ** | *** | ********* |
| Sonestedt, 2011 | **** | ** | *** | ********* |
| Selection column includes four items: (1) representativeness of the exposed cohort, (2) selection of the non-exposed cohort, (3) ascertainment of exposure to implants, and (4) demonstration that outcome of interest was not present at start of study. Comparability column includes two items: (1) study controls for important and (2) any additional covariates. Outcome column includes three items: (1) assessment of outcome, (2) follow-up long enough for outcomes to occur, and (3) adequacy of follow up. | | | | |

**Figure S1.** Funnel plot of the 14 comparatives.
